# OCT risk factors for 2-year foveal involvement in non-treated eyes with extrafoveal geographic atrophy and AMD

**DOI:** 10.1007/s00417-024-06399-9

**Published:** 2024-02-08

**Authors:** Enrico Borrelli, Costanza Barresi, Alessandro Berni, Pasquale Viggiano, Michele Reibaldi, Ugo Introini, Francesco Bandello

**Affiliations:** 1 Department of Surgical Sciences, University of Turin, Turin, Italy; 2 Department of Ophthalmology, “City of Health and Science” Hospital, University of Turin, Via Cherasco, 23, Turin, Italy; 3 Vita-Salute San Raffaele University, Milan, Italy; 4 IRCCS San Raffaele Scientific Institute, Milan, Italy; 5 Department of Basic Medical Sciences, Neuroscience and Sense Organs, University of Bari “Aldo Moro”, 70121 Bari, Italy

**Keywords:** Optical coherence tomography, Geographic atrophy, Age-related macular degeneration, Biomarkers

## Abstract

**Purpose:**

To assess the relationship of optical coherence tomography (OCT) findings and progression to foveal atrophy in a cohort of eyes with extrafoveal geographic atrophy (GA) and age-related macular degeneration (AMD) at inclusion.

**Methods:**

We retrospectively analyzed 45 participants (45 eyes) with extrafoveal GA at baseline and with 2 years of regular follow-ups. Several OCT qualitative features (i.e., presence of foveal flat pigment epithelium detachment with a thin double layer sign [DLS] and reticular pseudodrusen, GA focality) and quantitative measurements (outer retinal layer thickness, retinal pigment epithelium [RPE] to Bruch’s membrane [BM] volume, minimum distance from the central foveal circle, and untransformed GA lesion size area) were assessed at baseline. Logistic regression analyses were carried out to identify independent significant predictors and compute odds ratios (ORs) for the risk of the development of atrophy.

**Results:**

At month 24, 26 eyes (57.8%) developed atrophy in the foveal central circle, while 11 eyes (24.4%) developed atrophy in the foveal central point. Significant independent predictive features for the development of atrophy in the foveal central circle included foveal outer retinal thickness (OR, 0.867; *p* = 0.015), minimum distance from the foveal central circle (OR, 0.992; *p* = 0.022), and foveal thin DLS (OR, 0.044; *p* = 0.036). The only independent predictive feature for the development of atrophy in the foveal central point was the presence of foveal thin DLS (OR, 0.138; *p* = 0.017).

**Conclusions:**

We identified OCT risk factors for 2-year foveal atrophy in eyes with untreated extrafoveal GA at baseline.


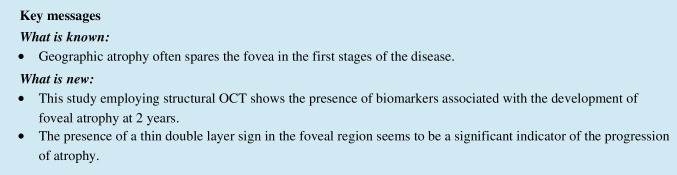


## Introduction

Geographic atrophy (GA) is the non-neovascular form of late age-related macular degeneration (AMD) [1]. GA is responsible for 10 to 20% of cases of legal blindness in patients with AMD [2] and it is estimated to affect more than 5 million people worldwide with a prevalence increasing exponentially with age [3].

Even though GA is a complex disease entity with multifactorial etiologies, this disorder is ultimately characterized by loss of the outer retina, retinal pigment epithelium (RPE), and choriocapillaris (CC) [4]. Although GA may affect the fovea at initial diagnosis, patches of atrophy typically involve the parafoveal macula at the beginning. This phenomenon, known as foveal sparing, characterizes a form of GA referred to as extrafoveal GA [5]. Of note, the term foveal-sparing GA is confined to extrafoveal GA cases with RPE atrophy surrounding the fovea by more than 270°. Previous studies have speculated that preferential foveal sparing may be secondary to the lower susceptibility of cone photoreceptors to cell death in the setting of AMD [6].

Importantly, assuming that GA regions are associated with corresponding absolute scotomas [7], central visual function typically remains unaffected until GA reaches the fovea [8]. Therefore, progression with respect to the distance to the fovea is a key determinant of visual prognosis in patients with extrafoveal GA at baseline. Of note, although the median time required for extrafoveal GA from first appearance to foveal center involvement is around 2.5 years [9], this period may be quite variable in clinical practice.

Assuming that the complement system appears to be involved in the GA pathogenesis and progression [10], complement inhibitors have been evaluated in clinical trials to understand whether these therapies are effective in slowing GA progression [11]. Following favorable results obtained in clinical trials [12], the use of an intravitreal complement C3 inhibition treatment (i.e., pegcetacoplan, SYFOVRE™, Apellis) was approved in February 2023 by the US Food and Drug Administration (FDA) for the treatment of GA. Intravitreal injection of pegcetacoplan proved to significantly slower GA progression as compared with sham treatment in AMD-associated GA [12]. Notably, pegcetacoplan-treated eyes were characterized by a significantly slower GA lesion progression toward the fovea [13]. Assuming the expected growing use of this drug and other complement inhibitors in the upcoming years, it would appear to be of great importance to detect risk factors for the foveal involvement in extrafoveal GA eyes at baseline. Importantly, the identification of these risk factors may broaden our knowledge regarding the AMD-associated GA pathogenesis.

Structural optical coherence tomography (OCT) is an essential diagnostic tool for the evaluation of individuals with GA as it provides anatomic details regarding the neuroretina and RPE. Previous reports have identified several OCT biomarkers associated with GA occurrence and progression. These biomarkers include the size, volume, and subtype of drusen, the presence of hyper-reflective foci, thin double-layer sign (DLS), and subretinal drusenoid deposits (i.e., also known as reticular pseudodrusen—RPD), thinning of the outer retina, photoreceptor degradation, choroidal thinning, and CC loss [4, 14–20].

In this longitudinal study over 2 years of follow-up, we explored the associations of structural OCT findings to progression to foveal atrophy in a cohort of eyes with extrafoveal GA at inclusion (study baseline).

## Methods

The San Raffaele Ethics Committee approved this retrospective cohort study. This study adhered to the 1964 Helsinki Declaration and its later amendments. Informed consent was gained from all individuals included in the study.

### Subjects

Patients with extrafoveal GA secondary to AMD [21] were identified from the medical records at San Raffaele Scientific Institute. The diagnosis of GA was established through a comprehensive approach involving fundus ophthalmoscopy, blue fundus autofluorescence (BAF), and OCT. Specifically, GA was identified when a hypopigmented area with visible choroidal vessels on fundus ophthalmoscopy corresponded to a hypoautofluorescent area on BAF, accompanied by corresponding RPE atrophy visualized on structural OCT. A diagnosis of extrafoveal GA was established when atrophy was not detected in the foveal region, as determined through multimodal imaging. At baseline visit, the following exclusion criteria were considered for the study eye: (i) prior history or evidence of macular neovascularization (MNV), including non-exudative cases; (ii) history of previous ocular surgeries including anti-vascular endothelial growth factor (VEGF) injections; and (iii) history or evidence of other retinal and optic nerve disorders. To be included, subjects were also required to not develop MNV throughout the 2-year follow-up and have at least two yearly retinal visits including structural OCT, BAF, and infrared reflectance (IR) images covering a study period of 2 years (24 months) after the baseline visit. The population fulfilling these criteria was the starting cohort for this analysis (*n* = 412 GA patients in our database).

Structural OCT, BAF, and IR imaging was performed with the Heidelberg Spectralis HRA + OCT device (Heidelberg Engineering, Heidelberg, Germany). The spectral domain OCT imaging session included 19 horizontal B-scans covering approximately a 5.5 × 4.5-mm area centered on the fovea and 6 radial linear B-scans captured with enhanced depth imaging (EDI) and centered on the fovea. Each B-scan was composed by 25 averaged OCT images. A minimum signal strength of 25 was required to the OCT images to be included, as recommended by the manufacturer [22].

### OCT grading

Baseline and follow-up structural OCT images were first reviewed for eligibility by an experienced and certified grader (EB).

Therefore, at baseline, eligible eyes (*n* = 45) were independently graded for qualitative features and quantitative metrics by two graders (CB and AB) who were masked as to the final eyes’ outcomes (i.e., see below for details). Of note, the baseline visit was the first visit with evidence of extrafoveal GA.

In details, OCT images at baseline were graded for qualitative features that were previously demonstrated to be associated with GA occurrence and progression:

- Pigment epithelium detachment in the Early Treatment Diabetic Retinopathy Study (ETDRS) grid central circle (dimensions: 1 mm diameter): OCT images were scrutinized for the presence of flat pigment epithelium detachments (PEDs) with a thin DLS. The DLS was classified as thin when a single zone of low to medium reflectivity was evident between the RPE and Bruch’s membrane [20].
- Reticular pseudodrusen: the presence or absence of RPD was assessed on the basis of multimodal imaging analyses, as previously described in details [23].
- Focality: the graders graded GA as unifocal vs. multifocal using structural OCT, IR, and BAF images.

Disagreements between graders in the qualitative grading were resolved by further debate and open adjudication to yield a single reading. The final decision was made by an experienced and certified grader (EB) whether the two graders were not able to reach a single consensus result.

At baseline, OCT images were also employed to obtain quantitative measurements.

- The Spectralis built-in software was used to measure the outer retinal layer (ORL) thickness. As in a previous study on GA, the ORL extended from the upper boundary of the outer plexiform layer (OPL) to the inner boundary of the RPE [19]. Therefore, the ORL was a combination of OPL and outer nuclear layer (ONL). OCT metrics were obtained across each of the ETDRS subfields: the central foveal circle with a 1 mm diameter, the inner circle subfield (dimensions: inner and outer radii of 0.5 and 1.5 mm), and the outer circle subfield (dimensions: inner and outer radii of 1.5 and 3.0 mm). Before computing the thickness and volume values, all B-scans were scrutinized by graders and segmentations were manually corrected. The final segmentation was also reviewed and eventually corrected by an experienced and certified grader (EB).
- Using the Spectralis built-in software, the RPE to Bruch’s membrane (BM) volume was also obtained by applying inner and outer segmentation boundaries at the RPE and RPE fit (estimated Bruch’s membrane position) levels, as previously showed [19, 20]. Of note, the RPE-RPE fit volume was also termed the drusen volume in previous studies [20]. Assuming that this space may contain both drusen and flat PEDs with a thin DLS (i.e., in absence of other types of PEDs), we felt the term RPE to BM volume was a more preferred term to indicate this metric [19].
- Minimum distance from the central foveal circle: using the built-in software caliper, the graders measured the distance to the nearest GA border using OCT and IR images.
- Untransformed GA lesion size area: the graders measured the GA size within the inner and outer circle subfields on IR images using the built-in software caliper.

Finally, the set of follow-up visits was graded for GA progression toward the fovea, as follows:

- Foveal central area involvement was graded as present if GA involved the ETDRS grid central circle (dimensions: 1 mm diameter) [8]. We felt the latter assessment might be clinically relevant as the decrease of the spared area in the central 1 mm is associated with a severe decline in visual acuity in GA patients [8]. This grading was made by employing multimodal imaging (i.e., structural OCT, IR, and BAF). In detail, for IR and BAF images, the ETDRS grid was centered on the fovea, identified through structural OCT. Subsequently, the GA was graded to involve the fovea through multimodal imaging. In details, this was determined by the presence of a hypoautofluorescent area affecting the central circle, which corresponded to a hyperreflective area on IR imaging and was identified as region of RPE atrophy using structural OCT.
- Foveal central point involvement was graded as being present whether the foveal center point on the cross-sectional OCT was involved by GA [24]. Multimodal imaging (i.e., structural OCT, IR, and BAF) was employed to assess the location of the foveal center point in correlation with the GA border.

### Study outcomes and statistical analysis

Statistical calculations were performed using Statistical Package for Social Sciences (version 23.0.0.0, SPSS Inc., Chicago, IL, USA).

To detect departures from normal distribution, a Kolmogorov–Smirnov test was performed for all quantitative variables. Mean, standard deviation (SD), and interquartile range (IQR) were computed for all quantitative variables. Student’s *t*-test and non-parametric Mann–Whitney *U* test were conducted to investigate differences in continuous variables between groups at baseline. Qualitative variables were compared using Fisher’s exact test. Wilcoxon signed-rank test was computed to compare visual acuity between baseline and follow-up visits.

Logistic regression analysis for each variable was first performed to determine whether each baseline feature was associated with the development of atrophy in the foveal central circle (dependent variable) at the 24-month follow-up visit. Those features with a *p* value inferior to 0.1 were successively included in the multivariable logistic regression analysis that was carried out to identify independent significant predictors and compute odds ratios (ORs) for the risk of the development of atrophy. Similarly, univariable and multivariable regression analyses were also computed considering the development of atrophy in the foveal central point at the 24-month follow-up visit as the dependent variable.

The agreement between graders in the qualitative grading was investigated using the unweighted *k* statistic test.

## Results

A total of 45 eyes from 45 patients (29 females) with extrafoveal GA secondary to non-neovascular AMD were finally included in our analysis. Mean ± SD age was 75.9 ± 7.8 years. Mean ± SD number of follow-up visits throughout the 24 months following the baseline assessment was 5.4 ± 1.4.

Among the cohort of 45 eyes with extrafoveal GA at baseline, 26 eyes (57.8%) developed atrophy in the foveal central circle by 24 months, while 11 eyes (24.4%) developed atrophy in the foveal central point within the follow-up period.

Overall, the BCVA was 0.19 ± 0.18 LogMAR (Snellen VA of ~ 20/32) at baseline and 0.31 ± 0.36 LogMAR (Snellen VA of ~ 20/40) at the 24-month follow-up visit (*p* < 0.0001). The final BCVA was 0.21 ± 0.12 LogMAR (Snellen VA of ~ 20/32) and 0.41 ± 0.40 LogMAR (Snellen VA of ~ 20/50) in eyes without and with atrophy in the foveal central circle at the last follow-up visit (*p* = 0.045). Similarly, the final BCVA was 0.22 ± 0.18 LogMAR (Snellen VA of ~ 20/32) and 0.60 ± 0.18 LogMAR (Snellen VA of ~ 20/80) in eyes without and with atrophy in the foveal central point at the last follow-up visit (*p* = 0.001).

### Baseline risk factors for development of foveal atrophy

The quantitative values and prevalence of qualitative biomarkers of interest among these 45 eyes at baseline are reported in Table 1.

**Table 1:** Baseline variables

GA lesion size area—inner ring subfield (mm^2^)	0.90 ± 1.24 (0.20–1.14)
GA lesion size area—outer ring subfield (mm^2^)	0.58 ± 1.60 (0.00–0.07)
Minimum distance from the foveal central circle (µm)	280.7 ± 232.8 (114.7–337.7)
Focality (multifocality), *n* (%)	20 (44.4%)
Reticular pseudodrusen, *n* (%)	24 (53.3%)
Foveal thin DLS, *n* (%)	11 (24.4%)
Outer retinal layer thickness—foveal central circle (µm)	107.0 ± 15.8 (95.5–123.7)
Outer retinal layer thickness—inner ring subfield (µm)	76.9 ± 15.3 (66.6–88.8)
Outer retinal layer thickness—outer ring subfield (µm)	68.5 ± 13.6 (59.9–79.1)
RPE to BM volume—foveal central circle (mm^2^)	0.02 ± 0.01 (0.01–0.02)

Quantitative values are expressed in mean ± SD (IQR). Qualitative values are reported as number of eyes (percentage)

*GA*, geographic atrophy; *n*, number of eyes; *DLS*, double layer sign; *RPE*, retinal pigment epithelium; *BM*, Bruch’s membrane

**Fig. 1:**
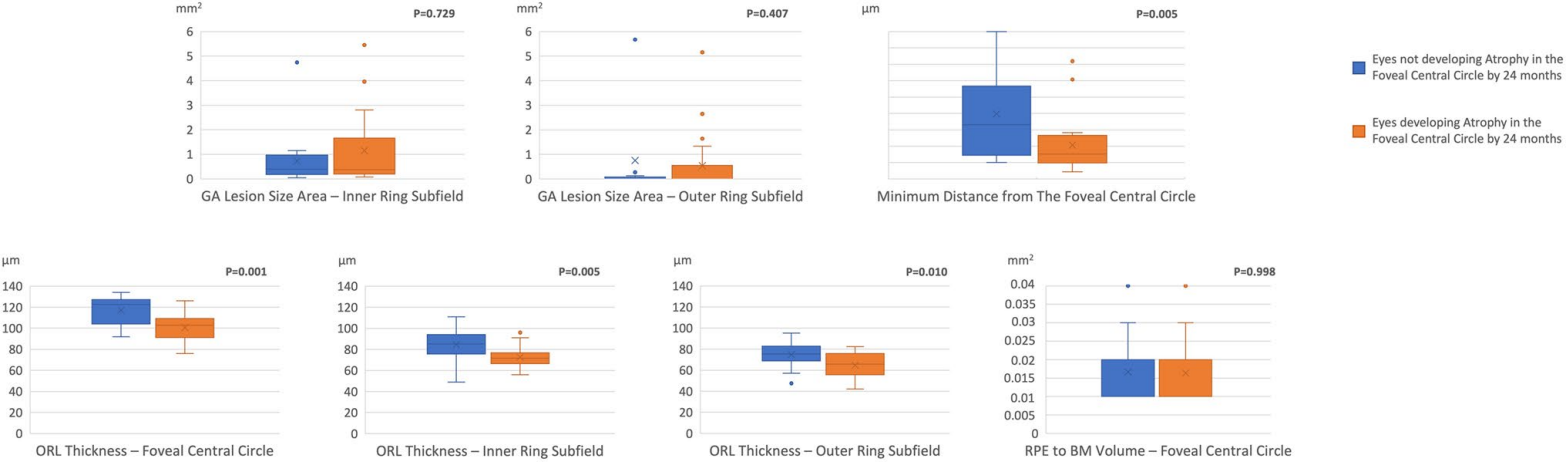
Box and whisker plots showing analyzed metrics in GA patients. Each box displays mean (cross within the box), median (central horizontal line), and interquartile range (horizontal extremes of the box) values for each metric. The ends of the whiskers illustrate the minimum and maximum values. Outliers are visualized as dots not included in whiskers. Each graph reports comparisons for a specific metric and *p* values for each comparison are reported

**Fig. 2:**
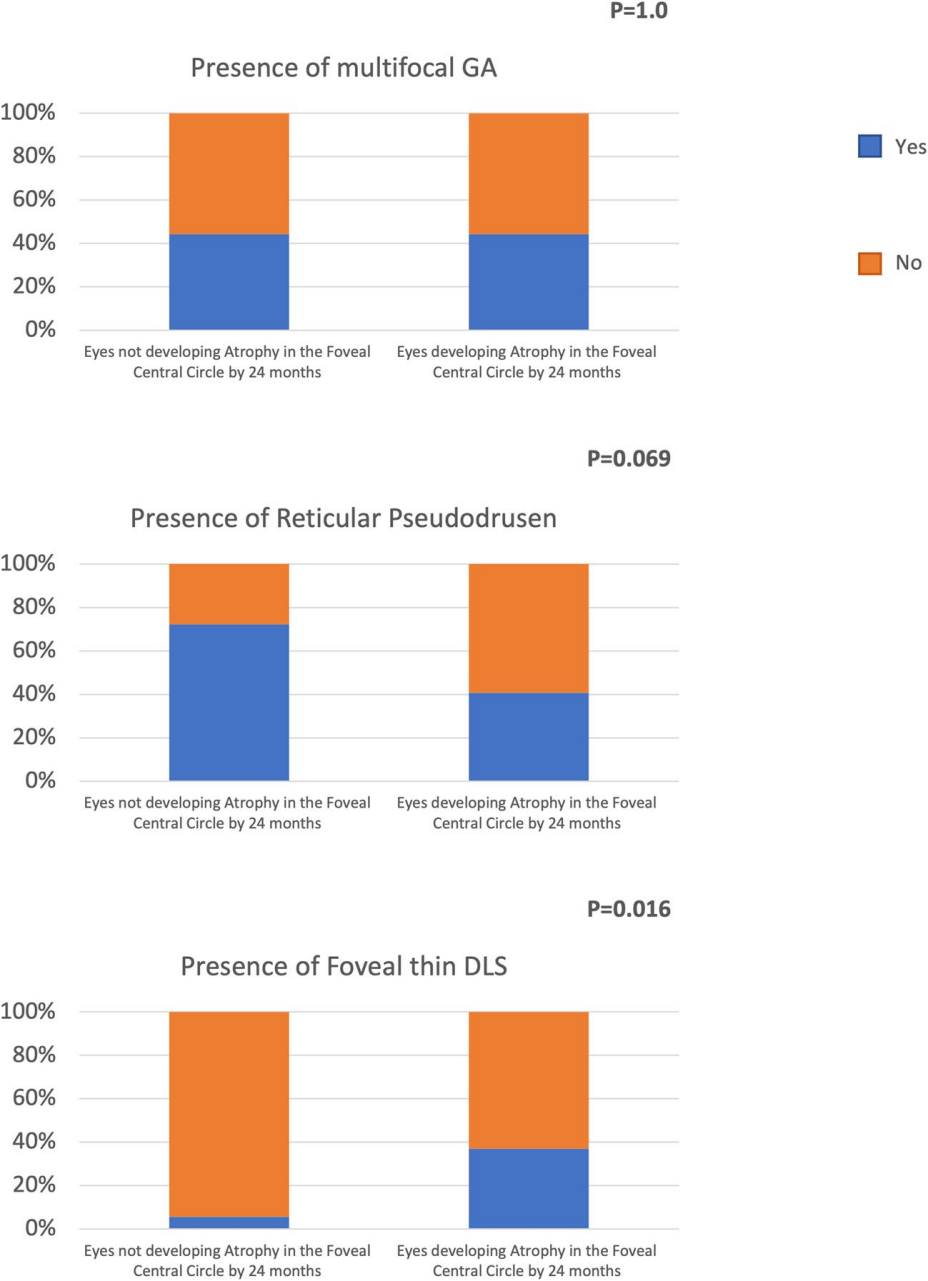
Grouped column chart showing the relative frequencies of qualitative findings in the study cohort. Each chart shows the relative frequencies of eyes graded with a specific finding. The relative frequencies are given as a percentage of patients with a specific qualitative finding in a distinct group (GA patients developing vs. not developing atrophy in the foveal central circle). On the X axis, columns are grouped on the basis of the development of atrophy in the foveal central circle. *p* values for each comparison are reported in the figure

**Fig. 3:**
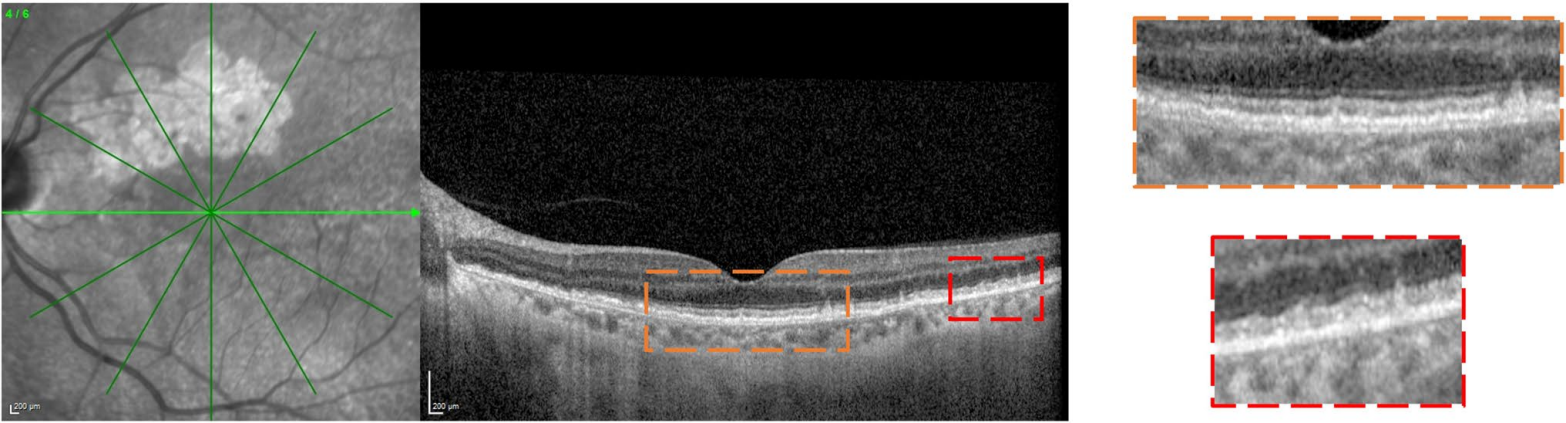
Representative optical coherence tomography (OCT) B-scan of a patient with extrafoveal GA. OCT B-scan (left) showing the presence of a thin DLS (orange dashed rectangle) and SDDs (red dashed rectangle). A magnified visualization of these regions of interest is reported on the right

At the baseline visit, the minimum distance from the foveal central circle and outer retinal thicknesses in all the analyzed subfields were lower in eyes developing atrophy in the foveal central circle within 24 months (Fig. 1). Figure 2 shows relative frequencies of each baseline imaging finding in the study cohort. The presence of foveal thin DLS was more prevalent in eyes developing atrophy in the foveal central circle within 24 months (Fig. 3).

Tables 2 and 3 summarize the results of the logistic regression analyses performed on the baseline imaging findings. Significant independent predictive features for the development of atrophy in the foveal central circle included the following: foveal outer retinal thickness (OR, 0.867; 95% confidence interval [CI], 0.772–0.972; *p* = 0.015), minimum distance from the foveal central circle (OR, 0.992; 95% CI, 0.986–0.999; *p* = 0.022), and foveal thin DLS (OR, 0.044; 95% CI, 0.002–0.812; *p* = 0.036) (Table 2). The only independent predictive feature for the development of atrophy in the foveal central point was the presence of foveal thin DLS (OR, 0.138; 95% CI, 0.027–0.697; *p* = 0.017) (Table 3).

**Table 2:** Baseline risk factors for the development of atrophy in the foveal central circle by 24 months

Baseline variables	Univariate analysis (logistic regression)			Multiple logistic regression		
	*Odds ratios*	*95% CI*	*p value*	*Odds ratios*	*95% CI*	*p value*
GA lesion size area—inner ring subfield	1.230	0.713–2.121	0.457	–	–	–
GA lesion size area—outer ring subfield	0.891	0.610–1.301	0.550	–	–	–
Minimum distance from the foveal central circle	0.996	0.992–0.999	0.015	0.992	0.986–0.999	0.022
Focality (multifocality)	1.071	0.320–3.585	0.911	–	–	–
Reticular pseudodrusen	3.545	0.974–12.905	0.055	1.415	0.165–12.106	0.751
Foveal thin DLS	0.094	0.011–0.821	0.032	0.044	0.002–0.812	0.036
Outer retinal layer thickness—foveal central circle	0.910	0.859–0.965	0.002	0.867	0.772–0.972	0.015
Outer retinal layer thickness—inner ring subfield	0.930	0.880–0.982	0.009	1.098	0.940–1.283	0.238
Outer retinal layer thickness—outer ring subfield	0.930	0.877–0.896	0.016	0.867	0.716–1.049	0.142
RPE to BM volume—foveal central circle	0.261	0.000–1.338E + 26	0.966	–	–	–

*CI*, confidence interval; *GA*, geographic atrophy; *DLS*, double layer sign; *RPE*, retinal pigment epithelium; *BM*, Bruch’s membrane

**Table 3:** Baseline risk factors for the development of atrophy in the foveal central point by 24 months

Baseline variables	Univariate analysis (logistic regression)			Multiple logistic regression		
	*Odds ratios*	*95% CI*	*p value*	*Odds ratios*	*95% CI*	*p value*
GA lesion size area—inner ring subfield	1.214	0.731–2.018	0.454	–	–	–
GA lesion size area—outer ring subfield	1.140	0.773–1.681	0.509	–	–	–
Minimum distance from the foveal central circle	0.999	0.996–1.003	0.692	–	–	–
Focality (multifocality)	0.607	0.149–2.475	0.487	–	–	–
Reticular pseudodrusen	2.692	0.655–11.061	0.170	–	–	–
Foveal thin DLS	0.149	0.033–0.681	0.014	0.138	0.027–0.697	0.017
Outer retinal layer thickness—foveal central circle	0.958	0.913–1.005	0.080	0.954	0.905–1.007	0.087
Outer retinal layer thickness—inner ring subfield	0.984	0.941–1.030	0.501	–	–	–
Outer retinal layer thickness—outer ring subfield	0.985	0.937–1.036	0.557	–	–	–
RPE to BM volume—foveal central circle	10,827.045	0.000–4.154E + 33	0.789	–	–	–

*CI*, confidence interval; *GA*, geographic atrophy; *DLS*, double layer sign; *RPE*, retinal pigment epithelium; *BM*, Bruch’s membrane

### Intergrader repeatability

The unweighted *k* values were 0.80 (42/45) for presence of foveal thin DLS, 1.0 (45/45) for presence of reticular pseudodrusen, and 1.0 (45/45) for GA focality.

## Discussion

In this study, we investigated baseline predictors of foveal involvement in subjects with extrafoveal GA and non-neovascular AMD at baseline. Overall, we demonstrated that several imaging risk factors are associated with a higher risk for progression from extrafoveal GA to foveal involvement after 2 years from the baseline, including lower foveal ORL thickness and minimum distance from the foveal central circle, and presence of foveal thin DLS.

The retina surrounding the atrophic area is known to be more prone to develop atrophy over time [25]. Therefore, the findings in our study of an increased risk to develop atrophy in the foveal central circle in eyes with a shorter minimum distance from the foveal central circle at baseline may indicate that early changes in the retina surrounding the atrophic lesion may be involving the fovea, this eventually leading to foveal atrophy over time. Baseline GA size and lesion number were not correlated with GA growth rate [26]. Consistently, our results did not show any association between foveal involvement after 2 years and these two characteristics at baseline.

In order to quantify the damage of the outer retina, we also performed a topographic quantitative analysis of the ORL thickness. In our analysis employing multivariate model, higher foveal ORL thickness at baseline was independently associated with a lower risk of foveal central circle within 2 years. In a previous important study, Zhang and colleagues [19] investigated whether the ORL thickness around GA may predict the annual enlargement rate of GA. They demonstrated that this thickness is inversely associated with annual GA growth. Therefore, our results appear to further confirm that the thickness of the outer retina in macular regions spared by GA is a consistent risk factor for atrophy involvement over time.

In our analysis, the presence of a foveal thin DLS was an independent baseline risk factor for GA involvement of the foveal central circle within 2 years. In AMD, it has been hypothesized that this OCT finding may correspond to regions of thick basal laminar deposit (BLamD) without vessels [19, 20, 27]. As proposed in previous studies [19, 20, 27], our finding that presence of a thin DLS is a risk factor for foveal atrophy within 2 years may be related to accumulation of BLamD obstructing perfusion of the RPE from the CC, which may further exacerbate the RPE ischemia and dysfunction. It is important to note that the presence of a foveal thin DLS was the only independent predictive feature for the development of atrophy in the foveal central point. In our study cohort, when the foveal central point becomes involved, a dramatic loss in vision occurs. Therefore, the definition of risk factors for the foveal central point atrophy may be clinically relevant. Since this was the only independent risk factor for the development of atrophy in the foveal central point, we may speculate that the presumed increase in basal laminar deposits in the foveal region may indicate a more consistent risk factor for the development of atrophy in this region.

Conversely, a thick DLS (i.e., flat PED enclosing more than 1 layer of low to medium reflectivity between the RPE and Bruch’s membrane) appears to be associated with presence of neovessels [20]. Of note, non-exudative neovessels in the AMD setting appear to provide support to the RPE and outer retina, this eventually leading to a protective effect against atrophy [28]. Assuming that non-exudative neovessels appear to be a well-established protective factor against RPE atrophy, though their presence may be relatively unusual, we did not include cases with this finding.

Reticular pseudodrusen (i.e., subretinal drusenoid deposits above the RPE on OCT and histopathology) may be commonly found in AMD eyes. Although RPD and drusen may coexist, these two abnormalities have significant differences in terms of histopathology and anatomy [29]. Importantly, while drusen are mainly confined to the foveal and parafoveal regions [30], RPD preferentially localize in the perifoveal macula and/or near-mid retinal periphery [31]. In our study cohort of eyes with extrafoveal GA at baseline, the presence of RPD was not associated with a greater risk for progression to foveal involvement within 2 years. Although the presence of RPD is a recognized risk factor for GA development and progression [32], we may speculate that this finding is not associated with a greater risk of foveal involvement as GA mainly expand into areas with RPD when present and RPD typically spare the foveal region [15]. It should be acknowledged, however, that the presence of RPD was not a protective factor for foveal atrophy in our study cohort. Future larger studies may elucidate whether the presence and distribution of RPD in extrafoveal GA eyes may impact on the progression of atrophy toward the fovea.

Several previous structural OCT studies have demonstrated that RPE to BM volume is a relevant risk factor for development and progression of GA in AMD patients [19, 20, 27, 33]. In a recent study, Chu et al. [14] performed a retrospective study with 38 eyes of 27 patients characterized by areas of GA. The authors demonstrated that RPE–BM distance around the GA area correlate with the annual enlargement of GA. As asserted above, a greater amount of material between the RPE and BM might exacerbate the outer retinal ischemia [19, 20, 27]. Alternatively, voluminous drusen may be more prone to collapse with resulting atrophy [34]. However, changes in drusen volume are dynamic and not progressive throughout the course of the disease. Therefore, drusen volume was suggested to not be a stable or consistent marker [20]. Consistently, in the present study, the presence of a higher central RPE to BM volume was not associated with a greater risk for progression to foveal involvement.

The main limitation of our study was that our study cohort was not part of a large multicenter trial and included subjects did not undertake regular follow-up visits with consistent intervals. Another important limitation is that segmentation errors occur frequently in AMD eyes, resulting in erroneous measurements [35]. In order to moderate the latter limitation, OCT B-scans were scrutinized for segmentation errors that were manually corrected by two graders. Moreover, the manually adjusted boundaries were successively revised by an experienced and certified grader (EB). Another limitation is that OCT angiography was not available for this analysis and we were not able to assess whether the CC perfusion is an additional risk factor for the 2-year foveal involvement. Future OCT angiography studies may clarify the latter aspect.

In conclusion, this study identifies OCT risk factors for 2-year foveal atrophy in eyes with untreated extrafoveal GA at baseline. Future larger studies are needed to confirm our findings. Assuming that pegcetacoplan-treated eyes are characterized by a significantly slower GA lesion progression toward the fovea [13], the identification of risk factors for progression toward the fovea is clinically relevant.
